# RNA interference-mediated targeting of DKK1 gene expression in Ishikawa endometrial carcinoma cells causes increased tumor cell invasion and migration

**DOI:** 10.3892/ol.2013.1439

**Published:** 2013-07-03

**Authors:** NUO YI, QIN-PING LIAO, ZHEN-HUA LI, BAO-JIANG XIE, YU-HONG HU, WEI YI, MIN LIU

**Affiliations:** 1 Department of Obstetrics and Gynecology, Beijing Ditan Hospital, Capital Medical University, Beijing 100015, P.R. China; 2 Department of Obstetrics and Gynecology, Peking University First Hospital, Beijing 100034, P.R. China; 3 Department of Surgery, Beijing International Studies University Hospital, Beijing 100024, P.R. China

**Keywords:** β-catenin, DKK1, endometrial carcinoma, invasion, migration, MMP14, RNAi

## Abstract

The Wnt signaling pathway plays an essential role in tumor invasion and migration. DKK1 functions as an important inhibitor of the pathway and represents a promising target for cancer therapy. The aim of the present study was to determine the role of DKK1 in endometrial carcinoma (EC) cell invasion and migration using RNA interference (RNAi) technology. Ishikawa EC cells were transfected at high efficiency with specific DKK1 siRNA. RT-PCR and western blot analysis were used to determine the mRNA and protein levels of DKK1, β-catenin and metalloproteinase 14 (MMP14) in siRNA-treated and -untreated cells. In addition, the invasion and migration of the EC cells were detected by invasion and migration assays. Transient transfection of DKK1 siRNA significantly inhibited the mRNA and protein levels of DKK1. Markedly increased cell invasion and migration was observed following treatment with DKK1 siRNA when compared with the negative control siRNA-treated and siRNA-untreated cells. The knockdown of DKK1 also elevated the mRNA and protein levels of β-catenin and MMP14 involved in the Wnt signaling pathway, indicating that targeting this gene may promote intracellular Wnt signal transduction and thus, accelerate EC cell invasion and migration *in vitro*. The RNAi-mediated targeting of DKK1 gene expression in Ishikawa EC cells resulted in increased tumor cell invasion and migration. DKK1 was identified as an inhibitor of EC cell invasion and migration via its novel role in the Wnt signaling pathway. Targeting DKK1 may therefore represent an effective anti-invasion and -migration strategy for the treatment of EC.

## Introduction

Endometrial carcinoma (EC) is a common malignant tumor of the female genital tract, which has notably increased in incidence over recent years (1). Tumor invasion and migration are characteristic features in the majority of malignant tumors, including EC (2,3). Previous studies have identified that abnormalities in the Wnt signaling pathway contribute to tumorigenesis in favor of tumor migration, invasion and metastasis (4–8). DKK1, an inhibitor of the Wnt signaling pathway, has been identified in the invasion and migration of specific benign and malignant tissues. β-catenin is a pivotal molecule in the Wnt signaling pathway and metalloproteinase 14 (MMP14) is a downstream target gene. In addition, these molecules have been identified as mediators of tumor invasion and migration. Therefore, in the current study, β-catenin and MMP14 were targeted using DKK1 siRNA to identify the effects of DKK1 on the invasion and migration of EC cells.

## Materials and methods

### Cell culture

Ishikawa EC cell lines were obtained from the American Type Culture Collection (Manassas, VA, USA). The cells were maintained in DMEM/F12 medium (Invitrogen Life Technologies, Carlsbad, CA, USA) supplemented with 10% fetal bovine serum (GE Healthcare, Amersham, UK), 100 μg/ml penicillin and 100 μg/ml streptomycin in a humidified atmosphere containing 5% CO_2_ at 37°C. Routine testing confirmed that the cells were free of mycoplasma and viral contaminants. The cells were subcultured every 2 days at a ratio of 1:2.

### Cell transfection

The following primer sequences for siRNAs targeting human *DKK1* were used: i) (RNA)-AUA GCG UUG GAA UUG AGA ACC GAG U; ii) (RNA)-ACU CGG UUC UCA AUU CCA ACG CUA U; and iii) (RNA)-AAU CCU GAG GCA CAG UCU GAU GAC C. Stealth™ RNAi Negative Control Med GC was used as a negative control for the siRNA (siRNA sequences were obtained from Invitrogen Life Technologies).

### Transfection conditions

The EC cells were transfected with DKK1 siRNA or negative control siRNA or untransfected (DKK1 RNAi, control and blank groups, respectively). The EC cells were then seeded in 35-mm culture dishes at 1×10^6^ cells/well prior to transfection with DKK1 siRNA or negative control siRNA using Lipofectamine 2000 reagent, according to the manufacturer's instructions. Lipofectamine 2000 (5 μl) diluted in 250 μl Opti-MEM was prepared. In addition, 10 μl DKK1 siRNA (20 μM) and 10 μl negative control siRNA (20 μM) were diluted with 250 μl Opti-MEM and incubated for 20 min. The 500 μl complexes of Lipofectamine 2000 and siRNA plus 1,500 μl DMEM/F12 were introduced to 35-mm culture dishes and incubated in a humidified atmosphere containing 5% CO_2_ at 37°C. After 5–6 h, the medium was replaced with 10% serum-supplemented DMEM/F12 and the cells were incubated for 24–96 h for further use in various procedures (all reagents were obtained from Invitrogen Life Technologies).

### Transfection efficiency

BLOCK iT™ fluorescent oligos (Invitrogen Life Technologies) were transfected into the cells of the DKK1 RNAi and control groups to ensure the successful transfection of siRNA into the cells.

### Silencing efficiency

The silencing efficiency was determined by RT-PCR and western blot analysis using DKK1-specific primers and antibodies. Subsequent experiments focused on the primer previously described as primer ii in the cell transfection methods for siRNAs targeting human DKK1*,* since it was identified as the most effective for inhibiting DKK1 expression.

### Semi-quantitative RT-PCR analysis

mRNA levels of DKK1, β-catenin, MMP14 and GAPDH (internal control) were determined by RT-PCR. Following cell incubation, total RNA was extracted from the cells using TRIzol^®^ reagent (Invitrogen Life Technologies). The reverse transcription reaction was set up using RT reaction mix (Promega Corporation, Madison, WI, USA) and the resultant cDNA was used for PCR. The following primers for DKK1, β-catenin, MMP14 and GAPDH were used: i) DKK1 sense, 5′-CTGCATGCGTCACGCTATGT-3′ and antisense, 5′-TCCTCGGAAATGATTTTGATCA-3′; ii) β-catenin sense, 5′-CGGGATGTTCACAACCGAAT-3′ and antisense, 5′-TTGGATGTTTTCAATGGGAGAA-3′; iii) MMP14 sense, 5′-CAGGGTCTCAAATGGCAACA-3′ and antisense, 5′-TTGCGAATGGCCTCGTATG-3′; and iv) GAPDH sense, 5′-CAGTCAGCCGCATCTTCTTTT-3′ and antisense, 5′-GTGACCAGGCGCCCAATAC-3′. Experiments were performed in triplicate.

### Western blot analysis

Protein levels of intracellular DKK1, active-β-catenin, MMP14 and β-actin (internal control) were detected by western blot analysis. When the cells reached 80–90% confluence, they were lysed in lysis buffer with protease inhibitors at 4°C. Cell lysates were also collected and protein concentrations were determined using the Bradford method. The lysates were cleared by centrifugation and quantified using the DC protein assay (Bio-Rad, Hercules, CA, USA). The protein samples (50 μg) were boiled for 5 min prior to being loaded onto 10% SDS-PAGE. Following electrophoresis, proteins were transferred onto a nitrocellulose membrane (Pall Corp., Washington, NY, USA). The membranes were blocked with 5% skimmed milk in PBS and probed with primary antibodies overnight at 4°C. Horseradish peroxidase-conjugated secondary antibodies (1:5,000; Santa Cruz Biotechnology, Inc., Santa Cruz, CA, USA) were used and the membranes were developed following an enhanced chemiluminescence detection protocol (Santa Cruz Biotechnology, Inc.). The following primary antibodies and dilutions with blocking solution were used: DKK1 (mouse monoclonal, 1:300; Abnova, Taipai City, Taiwan); active-β-catenin (mouse monoclonal, 1:500; Upstate Biotechnology, Lake Placid, NY, USA); MMP14 (rabbit polyclonal, 1:300; Abcam, Cambridge, UK) and β-actin (mouse monoclonal, 1:1,000; Santa Cruz Biotechnology, Inc.). All experiments were performed in triplicate.

### Invasion assay

An invasion assay was performed using the Transwell chamber assay according to previous studies (9–11), with modifications. Briefly, Matrigel matrix (1:10 v/v, 1 mg/ml; BD Biosciences, Franklin Lakes, NJ, USA) was diluted in serum-free DMEM/F12. Then, the diluted Matrigel matrix was addd to the upper wells of a 24-well transwell plate (polycarbonate membranes with an 8-μm pore size; Millipore, Billerica, MA, USA) and incubated for 5–6 h at room temperature. The cells of the DKK1 RNAi and blank groups were trypsinized, washed and resuspended. Viable cells were added to the upper wells at a density of 2×10^5^ cells/well with 300 μl serum-free DMEM/F12, whilst 500 μl serum-supplemented DMEM/F12 was filled into the lower wells. Plates were incubated at 37°C for 48 h, then non-invaded cells on top of the transwell were scraped off with a cotton swab. The cells in the chambers were maintained in an incubator at 37°C and allowed to migrate for 48 h. After 48 h, the non-migrated cells in the upper compartments were scraped carefully with cotton swabs. Migrated cells adhering to the lower surface of the membranes were fixed with methanol and stained with hematoxylin and eosin. The membranes were excised from the insert and mounted onto glass slides for light microscopic analysis, and migrated cells (penetrating Matrigel matrix and pores) were counted (magnification, ×20) from ten randomly selected fields. Each sample was assayed in triplicate.

### Migration assay

A migration assay was performed following the method for the invasion assay, with the exception of the exclusion of the Matrigel matrix and the use of polycarbonate membranes only.

### Statistical analysis

Data are presented as the mean ± SD. Statistical analyses were performed using SAS Version 9 (SAS Institute Inc., Cary, NC, USA). P<0.05 was considered to indicate a statistically significant difference. Quantitative analyses were performed using the Student's t-test for RT-PCR, western blot analysis and invasion and migration assay results among the three groups.

## Results

### Effect of DKK1 siRNA on DKK1 mRNA levels

**Figure 1:**
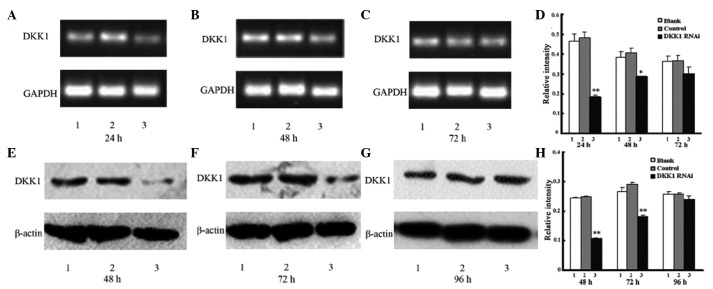
Effect of DKK1 siRNA on mRNA and protein levels of DKK1. Total cell extract levels of DKK1 mRNA in the three groups were measured by RT-PCR at (A) 24, (B) 48 and (C) 72 h. (D) Data are presented as density of DKK1/GAPDH. DKK1 protein levels of cell lysates were detected by western blot analysis at (E) 48, (F) 72 and (G) 96 h. (H) Data are presented as density of DKK1/β-actin. ^*^P<0.05 and ^**^P<0.001, vs. blank group. 1, blank; 2, control; and 3, DKK1 RNAi groups. RT-PCR, reverse transcription polymerase chain reaction.

The EC cells were transfected successfully with DKK1 and negative control siRNAs with high efficiency. DKK1 mRNA levels in total cell extracts were measured by semi-quantitative RT-PCR. Negligible changes in DKK1 mRNA levels were identified in the blank and control groups, however, in the DKK1 RNAi group, the DKK1 mRNA levels showed a decrease between 24, 48 and 72 h (61.47, 25.79 and 17.04%, respectively, vs. blank). In the DKK1 RNAi group, DKK1 gene expression was significantly inhibited between 24 and 48 h, however, there was no marked difference after 72 h. mRNA levels of GAPDH, as a loading control, were also analyzed, however, no changes were observed (Fig. 1 and Table I).

### Effect of DKK1 siRNA on *DKK1* protein level

The levels of DKK1 protein in DKK1 siRNA-treated cells were identified to significantly decrease between 48, 72 and 96 h (56.74, 31.64 and 7.17%, respectively, vs. blank group) when compared with the untreated and control-treated cells. Inhibition was identified as statistically significant between 48 and 72 h, however, no significant difference was identified after 96 h. No change in the protein levels of β-actin, which served as a loading control, were identified. In addition, no significant differences were identified in protein levels in the blank and control groups (Fig. 1 and Table II).

### Effect of DKK1 siRNA on EC cell invasion

**Figure 2:**
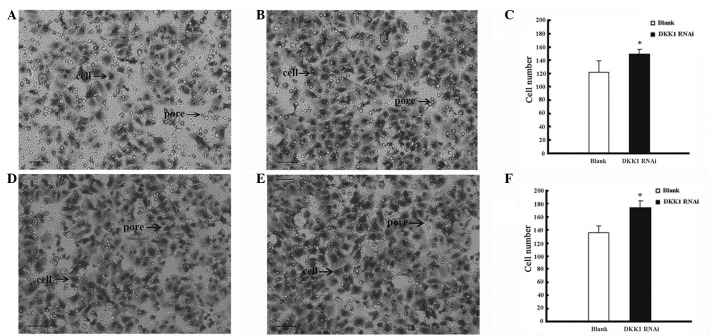
Invasion and migration assays were performed using the transwell chamber assay. Migrated cells stained with HE were counted by light microscope (magnification, ×20) from randomly selected fields. Invasion assay (A) blank and (B) DKK1 RNAi groups. (C) Transfection with DKK1 siRNA, average cell number (149.80) penetrating the Matrigel matrix and pores was significantly increased when compared with that of the blank group (122.50), and cell invasion was elevated by 22.29%. Migration assay (D) blank and (E) DKK1 RNAi groups. (F) Transfection of DKK1 siRNA, average cell number (173.90) penetrating the pores was significantly increased when compared with the blank group (135.80), and cell migration was elevated by 28.06%. ^*^P<0.05, vs. blank group. Bar indicates 50 μm. HE, hematoxylin and eosin.

Each field was observed under a light microscope (magnification, ×20) and a significant difference was identified between the number of migrated cells in the DKK1 RNAi group (149.80) when compared with the blank group (122.50), and the knockdown of DKK1 was identified to result in accelerated EC cell invasion (Fig. 2).

### Effect of DKK1 siRNA on EC cell migration

Cell migration numbers for DKK1 siRNA-treated and -untreated cells were 173.90 and 135.80, respectively. The results indicated that the cells treated with DKK1 RNAi had significantly accelerated EC cell migration when compared with the blank group (Fig. 2).

### β-catenin and MMP14 mRNA expression following knockdown of DKK1

**Figure 3:**
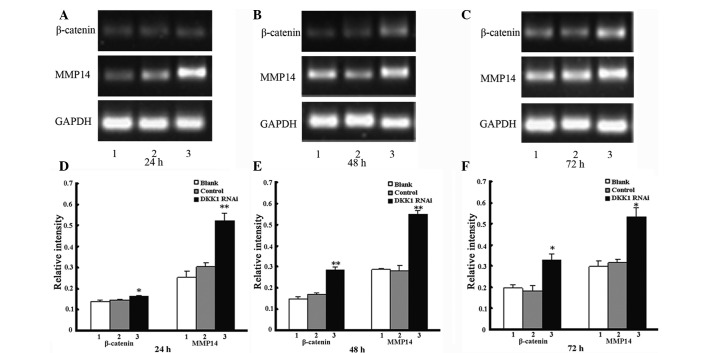
RT-PCR analyses of β-catenin and MMP14 mRNA expression in the three groups at (A) 24, (B) 48 and (C) 72 h. (D–F) Data are presented as density of β-catenin or MMP14/GAPDH. ^*^P<0.05 and ^**^P<0.001, vs. blank group. 1, blank; 2, control; and 3, DKK1 RNAi groups. MMP14, metallopeptidase 14; RNAi, RNA interference.

The mRNA levels of β-catenin and MMP14 were increased in the DKK1 siRNA-treated cells when compared with the untreated and control-treated cells after 72 h. In addition, the mRNA levels of β-catenin and MMP14 were significantly elevated following the knockdown of DKK1, however, no significant differences were identified in the blank and control groups (Fig. 3 and Tables III and IV).

### β-catenin and MMP14 protein expression following DKK1 knockdown

**Figure 4:**
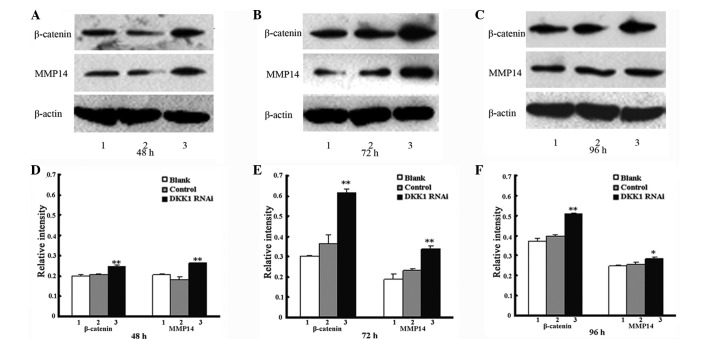
Western blot analyses of active-β-catenin and MMP14 protein expression in the three groups at (A) 48, (B) 72 and (C) 96 h. (D–F) Data are presented as density of β-catenin or MMP14/β-actin. ^*^P<0.05 and ^**^P<0.001, vs. blank group. 1, blank; 2, control and 3, DKK1 RNAi groups. MMP14, metallopeptidase 14; RNAi, RNA interference.

Western blot analyses of cell lysates were performed to analyze whether protein expression correlated with mRNA expression following the knockdown of DKK1. The protein levels of β-catenin and MMP14 in the DKK1 RNAi group were significantly elevated after 96 h when compared with that of the additional two groups (Fig. 4 and Tables V and VI), and no significant differences were identified in the protein levels of the blank and control groups.

## Discussion

EC is the most common malignant tumor in females and has a high incidence worldwide (1). The majority of EC cases are metastatic at diagnosis and therefore, metastasis is the main cause of cancer-related mortality. Tumor cell invasion is a complex event that involves interactions among tumor cells, extracellular matrix (ECM) degradation and cell migration (2). Cell migration and invasion are early steps in metastasis (3) and therefore it is necessary to identify specific molecules and proteins that may limit the process of cell invasion and migration.

Previous studies have identified that abnormalities in the Wnt signaling transduction pathway contribute to tumorigenesis involved in cell migration, invasion and metastasis (4–8). A number of molecules and proteins involved in the Wnt signaling pathway have been investigated as targets for the diagnosis and treatment of malignant tumors. The present study focused on DKK1, a negative regulator in the Wnt signaling pathway (12,13), as a key factor previously identified to be involved in the invasion and migration of colorectal (14), neuroblastoma (15) and placental cells (16,17).

β-catenin functions as a significant component in the Wnt signaling pathway, and interactions with frizzled and LRP5/6 receptors result in the dephosphorylation of β-catenin (non-phosphorylated active β-catenin). The dephosphorylated form of β-catenin has been shown to have significant effects on the Wnt signaling pathway (18,19). Subsequent to these interactions, accumulated active-β-catenin molecules translocate to the nucleus, activating downstream target genes, the majority of which, including the MMPs, are involved in tumorigenesis (20–23). The MMP family is comprised of zinc-dependent endopeptidases that are crucial for various proteolytic events and a number of tumor malignancy processes, including metastasis (24). MMPs are also involved in ECM degradation and therefore contribute to tumor progression and metastasis. Previous studies have shown that during tumorigenesis, MMPs are involved in tumor migration, metastasis and invasion into surrounding tissue. MMP14 is a member of the MMP family and has been identified to be involved in ECM degradation and invasion. In addition, the human DKK1 (chromosome 10q11.2) gene encodes an inhibitor involved in the Wnt signaling pathway, binding to and antagonizing LRP5/6 (25–27). In the present study, the hypothesized interactions between β-catenin, MMP14 and DKK1 in EC cell invasion and migration were investigated.

RNAi is a sequence-specific, post-transcriptional gene-silencing method initiated by double-stranded RNA and homologous to the gene being suppressed. RNAi is now routinely used for the transient knockdown of gene expression in a wide range of organisms for the analysis of gene function (28). In the present study, siRNA with high specificity and efficiency to DKK1 were used to suppress DKK1 gene expression in Ishikawa EC cells. In addition, DKK1, β-catenin and MMP14 were targeted using DKK1-targeting siRNA to identify the effects of DKK1 on EC cell invasion and migration. Few studies have analyzed the biology of DKK1 function in tumor invasion and migration.

In the present study, the transfection of DKK1 siRNA downregulated the mRNA and protein levels of DKK1 in the DKK1 RNAi group. DKK1 siRNA was identified to significantly inhibit DKK1 mRNA levels after 48 h, and protein levels were downregulated after 72 h. However, no marked changes in the levels of mRNA and protein were identified in the blank and control groups. These results demonstrated that the mRNA and protein levels of DKK1 were successfully downregulated by transfection of DKK1 siRNA, however, no significant decrease in the levels of mRNA and protein were identified in the DKK1 siRNA-treated cells after 72 and 96 h, respectively. This is hypothesized to be due to the transient nature of the DKK1-knockdown by DKK1 siRNA.

The knockdown of DKK1 has been identified to accelerate EC cell invasion and migration. In the present study, EC cells transfected with DKK1 siRNA showed increased tumor cell invasion and migration when compared with the untreated and control-treated cells, indicating an upregulation of β-catenin and MMP14. A significant increase in the mRNA and protein levels of β-catenin and MMP14 and cell invasion and migration were identified in the DKK1 RNAi group when compared with the additional two groups. The results of the current study are consistent to that of previous studies reporting the involvement of the knockdown of DKK1 in tumor cell invasion and migration (15–18). In addition, these were consistent with the results of our previous study, which demonstrated that EC cell invasion and migration may be inhibited by the upregulation of DKK1 (29–31).

Upregulation of β-catenin and MMP14 by the knockdown of DKK1 interferes with the Wnt signaling pathway and its downstream signaling events. Intracellular signaling transduction pathways are often exploited during tumor invasion and migration and the involvement of β-catenin and MMP14 in the Wnt signaling transduction pathway has been identified to accelerate tumor invasion and migration. Therefore, the hypothesized effects of the DKK1 RNAi-mediated upregulation of β-catenin and MMP14 on EC cell migration and invasion were investigated in the present study. A significant increase in the levels of β-catenin mRNA and protein in the DKK1 RNAi group was identified. The knockdown of DKK1 increased the levels of β-catenin dephosphorylation into active-β-catenin, which resulted in elevated activation of the Wnt signaling pathway. Accumulated active-β-catenin in the cytoplasm was translocated into the nucleus where it interacted with lymphocyte enhancers and T-cell transcription factors to activate downstream target genes, including MMP14. Marked increases in the MMP14 mRNA and protein levels in the DKK1 RNAi group indicated significant MMP14 activation. The results indicated that the upregulation of β-catenin and MMP14 the knockdown of DKK1 may result in the elevated activation of the Wnt signaling pathway and downstream signaling events involved in tumor migration and invasion.

In summary, the results of the present study indicate a novel biological function for DKK1 in the inhibition of EC cell invasion and migration. In addition, targeting DKK1 may represent an effective anti-invasion and -migration strategy for the treatment of EC, as DKK1 may contribute directly or indirectly to anti-tumorigenesis.

## Figures and Tables

**Table I:** DKK1 mRNA levels in the three groups at 24, 48 and 72 h.

Groups	24 h	48 h	72 h
Blank	0.465±0.039	0.385±0.027	0.363±0.029
Control	0.485±0.027	0.408±0.025	0.368±0.026
*DKK1* RNAi	0.179±0.013^a^	0.286±0.003^b^	0.301±0.035

Data are presented as density of DKK1/GAPDH.

a P<0.001 and

b P<0.05, vs. blank group.

**Table II:** DKK1 protein levels in the three groups at 48, 72 and 96 h.

Groups	48 h	72 h	96h
Blank	0.246±0.002	0.266±0.015	0.251±0.005
Control	0.250±0.002	0.292±0.006	0.258±0.006
*DKK1* RNAi	0.107±0.001^a^	0.182±0.003^a^	0.233±0.010

Data are presented as density of DKK1/β-actin.

a P<0.05, vs. blank group.

**Table III:** Active β-catenin mRNA expression following knockdown of DKK1 at 24, 48 and 72 h.

Groups	24 h	48 h	72 h
Blank	0.137±0.006	0.147±0.012	0.195±0.015
Control	0.144±0.002	0.169±0.006	0.182±0.024
*DKK1* RNAi	0.162±0.006^a^	0.283±0.016^b^	0.325±0.035^a^

Data are presented as density of β-catenin/GAPDH.

a P<0.05 and

b P<0.001, vs. blank group.

**Table IV:** MMP14 mRNA expression following knockdown of DKK1 at 24, 48 and 72 h.

Groups	24 h	48 h	72 h
Blank	0.252±0.029	0.287±0.005	0.296±0.026
Control	0.303±0.017	0.282±0.022	0.314±0.015
*DKK1* RNAi	0.162±0.006^a^	0.283±0.016^a^	0.532±0.044^b^

Data are presented as density of MMP14/GAPDH.

a P<0.001 and

b P<0.05, vs. blank group.

MMP14, metallopeptidase 14.

**Table V:** Active-β-catenin protein expression following knockdown of DKK1 at 48, 72 and 96 h.

Groups	48 h	72 h	96 h
Blank	0.200±0.005	0.301±0.004	0.373±0.014
Control	0.207±0.003	0.366±0.045	0.398±0.007
*DKK1* RNAi	0.244±0.007^a^	0.618±0.017^a^	0.510±0.005^a^

Data are presented as density of β-catenin/β-actin.

a P<0.001, vs. blank group;

b P<0.001, vs. blank group.

**Table VI:** MMP14 protein expression following knockdown of DKK1 at 48, 72 and 96 h.

Groups	48 h	72 h	96 h
Blank	0.206±0.005	0.188±0.026	0.247±0.005
Control	0.181±0.015	0.231±0.009	0.255±0.010
*DKK1* RNAi	0.262±0.003^a^	0.339±0.015^a^	0.285±0.006^b^

Data are presented as density of MMP14/β-actin.

a P<0.001, vs. blank group;

b P<0.05, vs. blank group.

MMP14, metallopeptidase 14.

## Abbreviations

ECM: extracellular matrix
RNAi: RNA interference
MMPs: metalloproteinases
EC: endometrial carcinoma
